# Roles of CircRNAs in Autoimmune Diseases

**DOI:** 10.3389/fimmu.2019.00639

**Published:** 2019-04-02

**Authors:** Xin Xia, Xinyi Tang, Shengjun Wang

**Affiliations:** ^1^Department of Laboratory Medicine, The Affiliated People's Hospital, Jiangsu University, Zhenjiang, China; ^2^Department of Immunology, Jiangsu Key Laboratory of Laboratory Medicine, School of Medicine, Jiangsu University, Zhenjiang, China

**Keywords:** non-coding RNA, circular RNA, property, function, autoimmune disease, biomarker

## Abstract

Circular RNAs (circRNAs) are covalently linked single-stranded RNAs, compared to linear counterparts that are relatively abundant, conserved, stable, and specific. Previously, most studies have revealed that circRNAs function in gene expression processes and participate in the pathogenesis of cancers, cardiovascular diseases, and neurological diseases. With advances in biotechnology, more biological functions of circRNAs have been found in several signaling pathways that are related to tumorigenesis, immunity, and metabolism. Recently, many circRNAs have been reported to be expressed abnormally and play important roles in the progression of autoimmune diseases. Thus, circRNAs may not only serve as potential biomarkers but also act as immune regulators and offer potential opportunities for therapy. This review briefly introduces the properties as well as the functions of circRNAs in different stages of gene expression. In addition, this article summarizes the available knowledge about abnormally expressed circRNAs in different autoimmune diseases and discusses their potential roles in these diseases, which helps us understand their regulatory mechanisms and provides future research perspectives.

## Introduction

Noncoding RNAs (ncRNAs), which are involved in gene transcriptional and post-transcriptional regulation, contribute to many human diseases (1–4). As an important member of ncRNAs, circular RNAs (circRNAs) have drawn great attention over recent decades. Distinct from linear RNA, a circRNA is a covalently linked single-stranded RNA without 5′ and 3′ ends (5). With the development of high-throughput RNA-seq technology and bioinformatics methods, some circRNA databases have been established. For example, circBase provides circRNA sequencing data. circ2Traits lists potential circRNAs associated with human diseases. circInteractome and circNet summarize the networks of RNA-binding protein (RBP)-circRNA and miRNA-circRNA.

Previously, circRNAs were considered miRNA sponges, and the primary focus of circRNA research was the circRNA-miRNA-mRNA network. However, an increasing number of studies have revealed the transcriptional and translational regulatory functions of circRNAs in various diseases, including several types of cancers (6), cardiovascular diseases (7, 8) and neurological diseases (9, 10). circRNAs can regulate proliferation, apoptosis, invasion, migration and cancer metastasis (11–14). Studies have proven that circRNAs can act as potential biomarkers for gastric cancer (15), colorectal cancer (16), and coronary artery disease (17). They also found that circRNAs participate in several pathways, such as tumor-related pathways, immune-related pathways and metabolism-related pathways. Recently, many studies have shown that circRNAs are abnormally expressed in human autoimmune diseases. In this review, we provide information about the expression profiles and analyze the functions as well as the research perspectives of circRNAs in autoimmune diseases.

## Properties of circRNAs

To date, a large number of circRNAs have been found. The majority of them have the following characteristics: (1) Abundance: circRNAs are abundant in diverse cell types and organisms (5). In some cases, the expression of circular forms of an RNA is even higher than that of the corresponding linear forms (18, 19). circRNAs can be transported by exosomes or microvesicles from the cell body to the extracellular fluid. More than 1000 circRNAs have been identified in human serum exosomes (20, 21). (2) Stability: compared to linear RNAs, circRNAs are more resistant to RNase due to their cyclical structure (19, 22). After treatment with actinomycin D, the half-lives of circRNAs still exceed 48 h, while the half-lives of the corresponding linear transcripts are <20 h (19, 23). (3) Conservation: circRNAs are present in divergent species, but in evolutionarily related species, such as humans and mice, 4% of orthologous genes can generate circRNAs (24), and ~5–30% of these circRNAs are completely conserved (25, 26). (4) Specificity: circRNAs often have specific expression profiles in different tissues and cells. They are involved in different developmental processes in certain diseases (27). circRNAs detected in the circulatory system can reflect the health condition of the distant tissue where they were produced. This suggests that disease-associated circRNAs are becoming more suitable diagnostic biomarkers and can assist in monitoring responses to interventions.

Based on their structures, there are three main classifications of circRNAs: (1) Exonic circular RNAs (ecircRNAs): They are formed by a reverse covalent connection between the 3′ splice donor of a downstream exon and the 5′ splice acceptor of an earlier or the same exon via intron pairing-driven circularization or lariat-driven circularization (28, 29). The main form of ecircRNA generation is intron pairing-driven circularization, which is also known as direct back-splicing (19, 24). After the flanking intronic complementary sequences of the pre-mRNA form a lariat, the introns will be eliminated. Lariat-driven circularization is also known as exon skipping (30). The RNA lariat formed by a pre-mRNA contains both exons and introns. When the introns between the circular exons are removed, ecircRNAs will be formed. Over 80% of circRNAs are ecircRNAs. (2) Intronic RNAs (ciRNAs): They are produced from the lariat introns of polymerase II (Pol II) transcripts linked by 2′5′-phosphodiester bonds at the joining sites and lack the 3′ linear sequence from the 3′ end of the intron to the branchpoint site. When there is a 7-nt GU-rich motif near the 5′ splice site and an 11-nt C-rich motif at the branchpoint site, these introns are not decomposed by the debranching enzyme; instead, they independently cyclize into ciRNAs (31). Intergenic circRNA is a special type of circRNA formed by two intronic circRNA fragments. The two fragments are flanked by GT-AG splicing signals at the circular junction (32). (3) Exon-intron circular RNAs (EIciRNAs): They are usually circularized by lariat-driven circularization containing exons and introns. The biogenesis of some EIciRNAs depends on RNA-binding proteins (RBPs). Through binding to the sequence motifs of upstream and downstream introns (33), RBPs promote flanking introns to become closer to each other. When they contain more than two exons, EIciRNAs can be stable. Otherwise, they are just formed as the intermediate step of processes that ultimately generate ecircRNAs by eliminating the introns. ecircRNAs predominantly exist in the cytoplasm, and ciRNAs and EIciRNAs are mainly located in the nucleus (34).

## Functions of circRNAs

**Figure 1:**
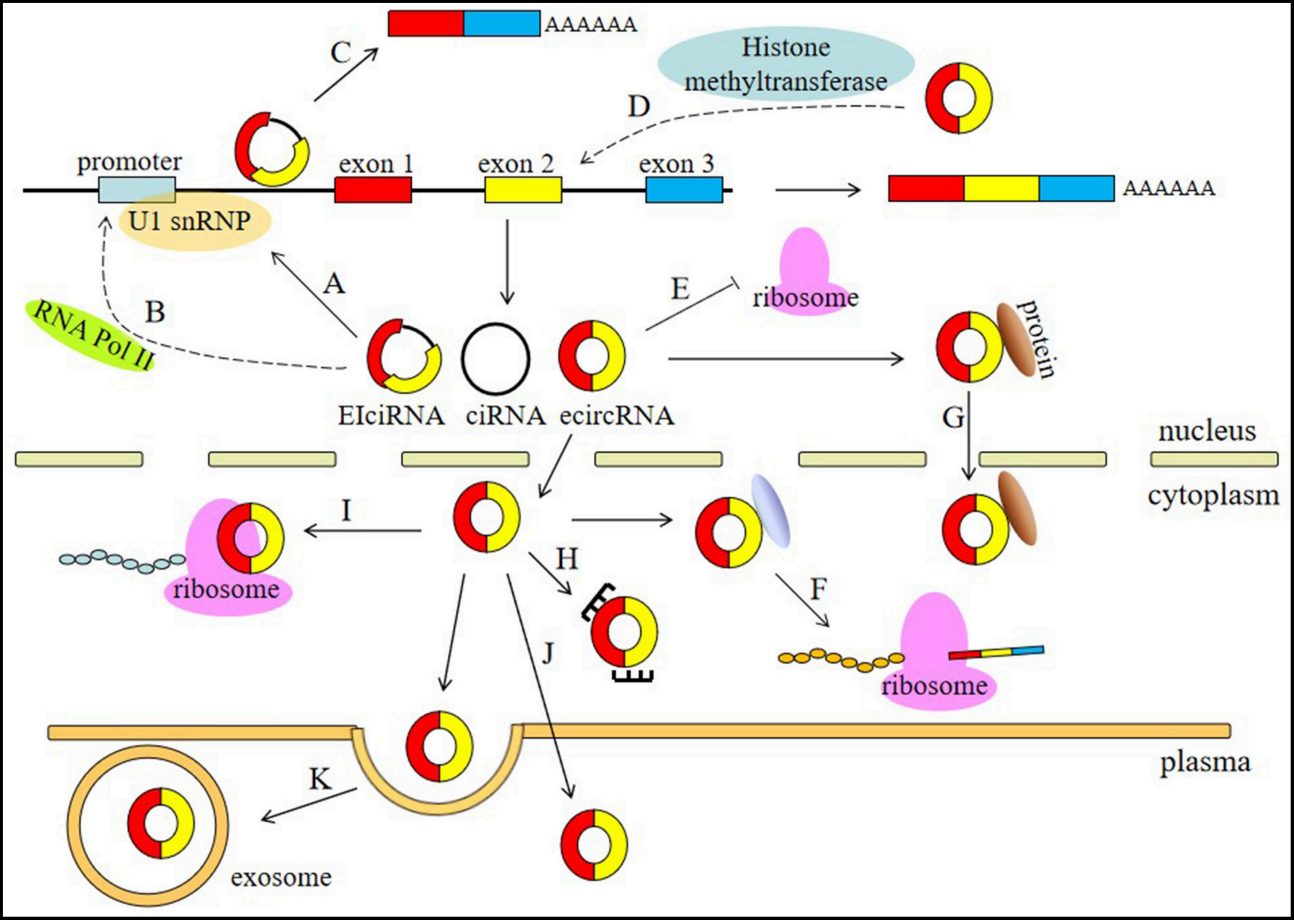
Functions of circRNAs. (A) circRNAs, especially EIciRNAs and ciRNAs, can interact with U1 snRNPs. (B) circRNAs are associated with the elongation RNA Pol II machinery. (C) circRNAs can contribute to the alternative splicing of their linear cognates. (D) circRNAs can participate in histone modification. (E) circRNAs can regulate RNA maturation. (F) circRNA can bind RBPs to regulate protein translation. (G) circRNA can regulate protein localization. (H) circRNAs can act as miRNA sponges. (I) circRNAs can be translated into proteins. (J) circRNAs can serve as biomarkers. (K) circRNAs can be transported by exosomes in the plasma.

circRNAs are involved in different physiological and pathological processes of human diseases and have a wide range of functions, including involvement in the processes of miRNA sponging, alternative splicing, RNAP II elongation, RNA maturation regulation, RBP sponging, protein localization, histone modification, and protein translation (Figure 1).

The most widely known circRNA function is serving as competitive endogenous RNAs (ceRNAs), which are also called microRNA (miRNA) sponges (26, 35). miRNA can bind to the matched 3′ UTR of mRNAs to mediate post-transcriptional silencing of protein-coding genes. Via competing for miRNA binding sites, circRNAs can indirectly regulate the expression of miRNA target mRNAs. ciRS-7 contains more than 60 putative miR-7 target sites and inhibits the binding of miR-7 to mRNA by sponging miR-7 to regulate the expression of miR-7 target mRNAs (36). Testis-specific circRNA sex determining region Y (Sry) contains 16 miR-138 binding sites (35). circHIPK3 can sponge miR-124 and miR-193 to regulate cell growth in cancer (37).

Transcriptional regulation is another widely known function and usually contributes to the alternative splicing of the linear cognates of circRNAs. EIciRNAs and ciRNAs that are restricted to the nucleus usually contribute to this type of regulation. For example, circSEP3 can bind to its cognate DNA locus via competing with a linear RNA, leading to the formation of alternatively spliced SEP3 mRNA with exon skipping. Notably, some circRNAs, especially those derived from exon 2, can sequester the translation start site, resulting in the production of non-coding linear transcripts. Additionally, circRNAs are associated with the elongation RNA Pol II machinery. circEIF3J and circPAOP2 increase transcription activity by interacting with U1 nuclear ribonucleoprotein (U1 snRNP) (33). ci-ankrd52 can accumulate at sites of transcription and positively modulate the efficiency of Pol II transcription to indirectly regulate the transcription of its parent gene (31).

circRNAs also impact protein translation control. circRNAs can impair the activity of rRNA-processing machinery to regulate RNA maturation so that protein translation will be slowed. circANRIL can decrease the interactions of the PeBoW complex member PES1 with pre-rRNA intermediates, which function in pre-mRNA processing during 60S ribosome maturation (38). As RBP sponges, circPABPN1 and circFoxo3 can inhibit proteins that are known to promote the translation of mRNAs. circPABPN1 can bind HuR, which is a well-studied RBP, and interact with multiple linear mRNAs, such as PABPN1 mRNA (39). As a result, the parent linear mRNA of circPABPN1 is affected, and less PABPN1 mRNA can be translated into protein. circFoxo3 is able to combine with CDK2 and p21 to inhibit the function of CDK2 (40). Apart from binding RBPs, circFoxo3 also regulates protein subcellular localization. circFoxo3 can interact with ID1 and E2F1 in the nucleus. The ectopic expression of circFoxo3, which is predominantly found in the cytoplasm, can facilitate the translocation of most ID1 and E2F1 in a pattern consistent with the distribution of circFoxo3 (41).

At the posttranslational level, circRNAs can participate in histone modification. cANRIL is a circular isoform produced from the non-coding transcripts of the INK4A-ARF-INK4B gene cluster (42). The p15^INK4B^ locus can be bound by polycomb repression complex 2 (PRC2), and histone H3 lysine27 (H3K27) trimethylation can repress the transcription of p15^INK4B^. cANRIL can bind and recruit PRC2 and is required for the occupancy of the p15^INK4B^ locus by PRC2 (43). Previous studies have indicated that non-coding ANRIL can bind CBX7, a H3K27me3-recognizing component of PRC1, and repress the INK4A and INK4B loci (44). This suggests that circRNAs may play a role in epigenetic control.

There is also a special process by which some circRNAs can be translated into functional polypeptides. Two typical circRNAs that go through this process are circZNF609 (45) and circMbl (46). There are at least two requirements for the circRNAs that are translated: one is that backsplicing should occur at the first exon. Another is that the 5′ UTR of the host gene should have properties similar to the internal ribosome entry sequence (IRES). These IRES-like sequences function independently of their orientation relative to the start codon (45). Although these polypeptides encoded by circRNAs have not yet been found to have fateful physiological functions, the stress conditions in which the splicing-dependent and cap-independent mechanism occurs can broaden our understanding of protein translation in extreme circumstances.

## circRNAs in Autoimmune Diseases

Normally, mechanisms of self-tolerance can protect an individual from self-reactive lymphocytes. When they fail, activation of self-reactive T or B cells will generate cell-mediated or humoral responses against self-antigens, leading to autoimmune diseases, such as rheumatoid arthritis (RA), systemic lupus erythematosus (SLE), multiple sclerosis (MS), and primary Sjögren's syndrome (pSS) (47, 48). Recent studies have reported that circRNAs are involved in autoimmune diseases (Table 1).

**Table 1:** Summary of circRNAs associated with autoimmune diseases.

**Disease**	**circRNA**	**Alias**	**circRNA classification**	**Position**	**Regulation**	**Fold change**	**Gene**	**Putative function**	**miRNA sponged**	**Target gene/pathway**	**References**
RA	hsa_circRNA_104871	–	Exonic	chr9	Upregulated	1.54	SUSD1	–	–	–	(49)
RA	hsa_circRNA_102902	hsa_circ_0057980	Exonic	chr2	Downregulated	0.18	PIKFVVE	miRNA sponge	miR−181d	–	(50)
RA	hsa_circRNA_104881	hsa_circ_0088088	Exonic	chr9	Downregulated	0.18	HSDL2	miRNA sponge	miR-16-5p	–	(50)
RA	hsa_circRNA_000931	hsa_circ_0001045	Intronic	chr2	Upregulated	2.7	MRPS5	miRNA sponge	miR-30a	BAFF	(50)
RA	–	hsa_circ_0044235	Exonic	chr17	Downregulated	0.5	CDC27	miRNA sponge	miR-892a	–	(51)
RA	ciRS-7	hsa_circ_0001946	Antisense	chrX	Upregulated	4.0	CDR1	miRNA sponge	miR-7 miR-671	PI3K/AKT	(52)
SLE	hsa_circRNA_400011	hsa_circ_0092374	Intronic	chr1	Upregulated	4.8	GADD45A	miRNA sponge	miR-296-3p miR-146b-3p miR-181d-3p miR-504-3p miR-328-5p	–	(53)
SLE	hsa_circRNA_102584	hsa_circ_0003146	Exonic	chr19	Upregulated	7.1	EHD2	miRNA sponge	miR-766-3p miR-762 miR-412-3p let-7i-3p miR-431-3P	–	(53)
SLE	hsa_circRNA_101471	hsa_circ_0034398	Exonic	chr15	Upregulated	6.5	C15orf41	miRNA sponge	miR-136-5p miR-665 miR-486-3p miR-601 miR-30b-3p	–	(53)
SLE	hsa_circRNA_100226	hsa_circ_0005567	Exonic	chr1	Downregulated	0.37	EPS15	miRNA sponge	miR-138-5p miR-145-3p miR-24-3p miR-620 miR-875-3p	–	(53)
SLE	hsa_circRNA_102165	hsa_circ_0045272	Exonic	chr17	Downregulated	0.5	ERN1	miRNA sponge	miR-6127	PAX8 DTX4	(54)
SLE		hsa_circ_0077179	Exonic	chr6	Downregulated	0.12	IBTK	miRNA sponge	miR-29b	AKT	(55)
MS	hsa_circRNA_101539	hsa_circ_0005402	Exonic	chr15	Downregulated	0.16	ANXA2	miRNA sponge	miR-1248 miR-766	–	(56)
MS	hsa_circRNA_101541	hsa_circ_0003452_2	Exonic	chr15	Downregulated	0.24	ANXA2	–	–	–	(56)
MS	–	hsa_circ_0106803	Exonic	chr17	Upregulated	2.8	GSDMB	miRNA sponge	miR-1275 miR-149	ASIC1	(57)
LN	hsa_circHLA-C	–	–	chr15	Upregulated	2.72	HLA-C	miRNA sponge	miR-4739 miR-6825-5p miR-6831-5p miR-6756-5p miR-3916	NF-κB	(58)
LN	hsa_circRNA_002453	–	–	–	Upregulated	5.6	RAD18	–	–	–	(59)
PBC	–	hsa_circ_402458	Exonic	chr2	Upregulated	1.86	CCNYL1	miRNA sponge	miR-522-3p miR-943	TGF-β	(60)

### Rheumatoid Arthritis

Studies have revealed that ~600 circRNAs are differentially expressed in the peripheral blood mononuclear cells (PBMCs) of RA patients compared with those of healthy controls (50). Among them, hsa_circ_0057980, hsa_circ_0088088, and hsa_circ_0001045 showed a close correlation with the progression of RA. As sponges of miRNAs, circRNAs contain several corresponding miRNA response elements (MREs). The level of miR-181, which is one of the hsa_circ_0057980 MREs, was significantly increased in RA patients. The level of miR-16-5p, which is one of the hsa_circ_0088088 MREs, correlated with the Th17/Treg cell imbalance and the degree of disease activity, such as the erythrocyte sedimentation rate (ESR), C-reactive protein (CRP) level and disease activity score (DAS28). miR-30a, which is one of the hsa_circ_0001045 MREs, could reduce cell apoptosis and negatively regulate BAFF synthesis in RA (50). Theoretically, these observations enhance the possibility that these circRNAs participate in the regulation of RA. hsa_circRNA_104871 and hsa_circ_0044235 have no significant correlation with disease severity measurements, including DAS28, ESR, CRP, rheumatoid factor (RF) and health assessment questionnaires (HAQ). However, ROC curve analysis showed their diagnostic value and found that hsa_circRNA_104871 and hsa_circ_0044235 could serve as useful biomarkers for RA (49, 51). miR-892a, which is one of hsa_circ_0044235 MREs, is proven to be increased in RA. It could target cytochrome P450 1A1 (CYP1A1) to mediate posttranscriptional repression, which is involved in the metabolism of carcinogenic metabolites to promote cell proliferation and invasion. Besides, ciRS-7 was also upregulated in RA peripheral blood compared with healthy controls and positively correlated with the level of anti-CCP (52).

### Multiple Sclerosis

MS is a common immune-mediated demyelinating disease of the CNS. Relapsing-remitting multiple sclerosis (RR-MS), accounting for almost 85% of MS cases, is the most prevalent type. Over 400 circRNAs are differentially expressed in the PBMCs of RR-MS patients compared with those of healthy controls. Hsa_circ_0106803, which is an alternative splicing abnormality of the GSDMB gene, exhibits a 2.8-fold expression upregulation in PBMCs in RR-MS (57). As predicted by the PITA algorithm, Hsa_circ_0106803 has more than 2 miRNA targets, such as miR-1275 and miR-149, which can contribute to susceptibility to MS (57, 61, 62). It was reported that miR-1275 can promote cell migration, invasion and proliferation. miR-149 can bind to ASIC1, which is a key subunit determining acid-activated currents in neurons, and reduce ASIC1 expression. Therefore, hsa_circ_0106803 might regulate the expression of ASIC1 by sponging miR-149 to modulate the progression of MS. In addition, two circRNAs from ANXA2, hsa_circ_0005402 and hsa_circ_0003452_2, are underexpressed in the PBMCs of patients. ANXA2 is reported to be a target of miR-155, whose expression increases greatly in PBMCs and correlates with disease severity in MS patients (63). Hsa_circ_0005402 shares 14 common miRNA targets with hsa_circ_0003452_2, suggesting cooperative regulation of the circRNA-miRNA-mRNA axis (56).

### Systemic Lupus Erythematosus

There are over 200 differentially expressed circRNAs in the plasma of SLE patients compared with that of normal controls. These circRNAs are widely and evenly distributed on all chromosomes, including the X chromosome. The upregulated circRNAs are mainly encoded on chr6 (8.85%), chr9 (7.96%), chr19 (7.96%), chr11 (7.08%), and chr3 (7.07%), while the downregulated circRNAs were mostly found on chr16 (10.64%), chr1 (8.51%), chr2 (8.51%), and chr7 (8.51%). According to their localization, we can speculate on the possible mechanisms of circRNAs. Four circRNAs, hsa_circ_0092374, hsa_circ_0003146, hsa_circ_0034398, and hsa_circ_0005567, are significantly dysregulated in the plasma according to validation experiments (53). It is helpful to understand the network of transcriptome regulation in SLE. In addition, there are approximately 127 differentially expressed circRNAs in the PBMCs. Hsa_circ_0077179, termed circIBTK, is downregulated and inversely correlated with the Systemic Lupus Erythematosus Disease Activity Index (SLEDAI) score and anti-dsDNA titer in patients. circIBTK expression also is positively correlated with the complement C3 levels. The expression of circIBTK was notably elevated when patients achieved significant clinical improvement. Since the AKT signaling pathway may account for the lymphocyte alteration in SLE (64), an additional study demonstrated that circIBTK could induce DNA methylation and regulate the AKT signaling pathway via miR-29b to regulate the proliferation and apoptosis of CD4+ T cells (55, 65). ROC curve analysis suggests that circIBTK might act as a biomarker and therapeutic target in SLE. In addition, hsa_circ_0045272 is downregulated in the T cells of SLE patients. After hsa_circ_0045272 expression knockdown in Jurkat cells, interleukin-2 production and the early apoptosis was upregulated. Apart from sponging miR-6127, hsa_circ_0045272 can regulate the transcription factors PAX8 and DTX4 at the mRNA level (54). PAX8 protein can inhibit apoptosis, and DTX4 protein can mediate the regulation of TANK-binding kinase, which is implicated in type I interferon production (66, 67), but their mRNA functions independent of protein require experimental verification.

### Lupus Nephritis

Lupus nephritis (LN) is a frequent manifestation of SLE triggered by glomerular immune complexes. LN class IV, which has the highest risk of end-stage renal disease, is the most common type of LN. Plasma hsa_circRNA_002453 is greatly increased in LN patients compared with RA patients, SLE without LN patients and healthy individuals. Its expression is positively correlated with 24 h proteinuria and the renal SLEDAI score, which are associated with the severity of renal involvement (59). A ROC analysis showed that hsa_circRNA_002453 can serve as a potential biomarker to distinguish LN from other autoimmune diseases. In addition, hsa_circHLA-C, which is greatly upregulated in LN renal biopsies compared with normal control kidneys, is positively correlated with traditional clinical indices of disease activity (58). A further GO enrichment analysis showed that the upregulated circRNAs might participate in several biological processes, such as dendritic cell (DC) differentiation, peptide antigen binding and cytoplasmic mRNA processing body assembly. A KEGG analysis indicated that the upregulated circRNAs modulate the HIF-1 and neurotrophin signaling pathways associated with the activation of NF-κB signaling in LN. This suggests that hsa_circHLA-C, which can participate in the NF-κB signaling pathway, can induce immune tolerance by regulating DCs. Additionally, hsa_circHLA-C can interact with miR-150. A previous study reported that renal miR-150 could upregulate profibrotic molecules by downregulating the expression of the antifibrotic protein suppressor of cytokine signaling 1 (SOCS1) to promote renal fibrosis. miR-150 decreased significantly in LN patients with low chronicity index (CI) scores (CI < 4) and increased in LN patients with high CI scores (CI ≥ 4) (68). miR-150 expression changes along with the changes in LN disease activity and the status of the treatment, indicating that upregulated hsa_circHLA-C might bind miR-150 at the onset of LN class IV disease to promote active kidney damage. The varying roles of circRNAs in different stages of diseases prompt us to consider disease activity and treatment status when we study circRNAs.

### Primary Biliary Cholangitis

Ursodeoxycholic acid (UDCA) is the current first-line therapy for primary biliary cholangitis (PBC). There are 22 circRNAs differentially expressed in the plasma of PBC patients vs. that of healthy individuals. In a study on the influence of UDCA treatment, hsa_circ_402458 was selected as a biomarker because its expression was significantly higher in PBC patients who were not receiving therapy than those receiving therapy (60). The ROC analysis showed that hsa_circ_402458 had the diagnostic value with the highest sensitivity and specificity for PBC among the candidates. In addition, hsa_circ_402458 is predicted to target two miRNAs, miR-522-3p and miR-943, which are involved in the abnormal resolution of inflammation and TGF-β pathway, respectively. Therefore, hsa_circ_402458 may function as an miRNA sponge to regulate inflammation-related pathways, contributing to the pathogenesis and development of PBC.

## Conclusions

Based on the present studies, the majority of circRNAs found in autoimmune diseases are ecircRNAs, and a few are ciRNAs and EIciRNAs. As circRNAs have close relationships with autoimmune diseases, the combined detection of different circRNAs and transitional markers may improve the efficiency of clinical diagnosis. Besides, circRNAs might be artificially synthesized for achieving miRNA loss-of-function *in vitro*. Recently, synthetic scRNA21 was verified to sponge miR-21 and increase the expression of miR-21 downstream proteins, while successfully suppressing gastric carcinoma cell proliferation (69). This provides a potential strategy for seeking therapeutic targets in the future. However, there are some notes which require additional attention. circRNA-based therapy should be delivered to the specific target cells which are still unknown. Considering that circRNAs function as miRNA sponges and RBP sponges, circRNAs often have several targets, which makes ceRNA networks more complicated and enhances the difficulty in improving curative effect of circRNA-based therapy. Although many circRNAs are under investigation, their roles in autoimmune diseases remain elusive. Advances in the methods, complement of circRNA databases and further study of circRNAs will be crucial to determine their mechanisms in autoimmune diseases.

## Author Contributions

All authors listed have made a substantial, direct and intellectual contribution to the work, and approved it for publication.

### Conflict of Interest Statement

The authors declare that the research was conducted in the absence of any commercial or financial relationships that could be construed as a potential conflict of interest.
